# Recurrent Urinary Tract Infections in Women: Current Management Guidelines and Therapeutic Options

**DOI:** 10.7759/cureus.114267

**Published:** 2026-08-10

**Authors:** Julia Sochowska, Agnieszka Buczkowska, Julia Danieluk, Natalia Adamaszek, Michal Glinski, Urszula Kacprzak, Laura Stochaj, Michalina Flejszman, Justyna Wojcik, Dominika Dolega-Mostowska

**Affiliations:** 1 Pulmonology, Allergology, and Pulmonary Oncology, Uniwersytecki Szpital Kliniczny w Poznaniu, Poznań, POL; 2 Endocrinology, Metabolism, and Internal Medicine, Uniwersytecki Szpital Kliniczny w Poznaniu, Poznań, POL; 3 Day Surgery Unit, Uniwersytecki Szpital Kliniczny w Poznaniu, Poznań, POL; 4 Orthopaedics, Traumatology, and Musculoskeletal Oncology, Uniwersytecki Szpital Kliniczny w Poznaniu, Poznań, POL; 5 Hypertension, Internal Medicine, and Metabolic Disorders, Uniwersytecki Szpital Kliniczny w Poznaniu, Poznań, POL; 6 Internal Medicine, Diabetology, Cardiology, and Nephrology, Szpital Wojewódzki w Poznaniu, Poznań, POL; 7 Medicine School, Uniwersytet Medyczny im. Karola Marcinkowskiego w Poznaniu, Poznań, POL; 8 Arterial Hypertension, Internal Medicine, and Metabolic Disorders, Uniwersytecki Szpital Kliniczny w Poznaniu, Poznań, POL; 9 Medicine School, Medical University of Lodz, Łódź, POL

**Keywords:** antimicrobial stewardship, eau guidelines, immunoactive prophylaxis, non-antibiotic prophylaxis, recurrent urinary tract infection

## Abstract

Recurrent urinary tract infections (rUTIs) are a common clinical problem affecting women worldwide. These infections significantly impair the quality of life, impacting social and sexual relationships, self-esteem, and work productivity. The escalating global threat of antimicrobial resistance necessitates a shift from repetitive antibiotic use toward effective non-antimicrobial preventive strategies. The objective of this literature review is to summarize the latest diagnostic and management guidelines (2023-2026) for rUTI in women, with a particular focus on comparing recommendations from international (European Association of Urology, American Urological Association/Canadian Urological Association/Society of Urodynamics, Female Pelvic Medicine & Urogenital Reconstruction) and Polish (Polish Urological Association, Polish Society of Gynecologists and Obstetricians, Polish Society of Family Medicine) scientific societies. A literature review was performed based on current guidelines and recent clinical studies. The literature selection involved databases such as PubMed/MEDLINE, EMBASE, the Cochrane Central Register of Controlled Trials (CENTRAL), Scopus, and Google Scholar. This review summarizes current recommendations for the diagnosis, prevention, and management of rUTIs in women. Antibiotic and non-antibiotic preventive strategies are discussed, including vaginal estrogen, methenamine hippurate, immunoactive prophylaxis, D-mannose, and cranberry products. Differences and similarities between international and Polish guidelines are highlighted. Management of rUTI requires a personalized, stepwise approach prioritizing behavioral modifications and non-antibiotic interventions. Clinicians must balance treatment efficacy with antimicrobial stewardship to mitigate collateral damage to the host microbiome.

## Introduction and background

Recurrent urinary tract infections (rUTIs) represent one of the most significant challenges in contemporary urology and gynecology, affecting millions of women globally and imposing a staggering burden on healthcare systems [1-3]. It is estimated that approximately 50-60% of women will experience at least one urinary tract infection (UTI) episode during their lifetime, with nearly 25-30% developing a recurrent form within six months of the initial event [4-6]. According to the harmonized definitions provided by the European Association of Urology (EAU) and American Urological Association (AUA), rUTI is diagnosed upon the occurrence of at least two documented symptomatic episodes within six months or three within one year [1,7].

While the majority of isolated infections are classified as uncomplicated, their cyclical nature drastically diminishes the patient’s health-related quality of life [4,8]. This leads to frequent work absenteeism, sexual dysfunction, and mounting “fear and frustration” associated with the anticipation of subsequent recurrences [5,9]. The economic dimension is equally profound: in the United States alone, annual costs are estimated at $3.5 billion. Moreover, in the United States, it is estimated that 11.3 million women experience UTIs annually, accounting for 7 million outpatient visits, 2 million emergency room visits, and 350,000 hospitalizations [10].

Furthermore, rUTIs are a primary driver for community antibiotic prescriptions (15-25% of the total) [3]. Given the “dry” antibiotic pipeline and the global rise of antimicrobial resistance (AMR), the latest clinical guidelines (2024-2026) place a strong emphasis on antimicrobial stewardship [11,12]. This review aims to provide a synthetic overview of current therapeutic options in the light of the most recent clinical evidence for clinicians involved in the care of women with rUTIs, including urologists, gynecologists, and primary care physicians [5,11]. The comparison of international and Polish guidelines aims to identify areas of agreement and differences in recommendations, taking into account the specific aspects of healthcare organization and clinical practice in Poland [11].

Epidemiology and etiopathogenesis

The incidence of rUTIs is notably high, with 26% of women experiencing a second infection within six months of their initial episode [13]. The prevalence follows a bimodal distribution, peaking during the reproductive years, often related to sexual activity, and increasing again after menopause due to estrogen deficiency [9,13]. The etiopathogenesis of rUTI is predominantly driven by uropathogenic Escherichia coli (UPEC) [14]. The high recurrence rate is largely attributed to the sophisticated survival and colonization mechanisms of UPEC [15]. Central to this process is the expression of fimbrial adhesins, which allow pathogens to bind specifically to the mucosal-epithelial surface of the host [15]. This stable attachment not only prevents the bacteria from being washed away by urine flow but also triggers bacterial internalization into the host cells [3,15]. Once inside, UPEC can replicate and aggregate to form intracellular bacterial communities (IBCs) [5,15]. These biofilm-like structures serve as a “dormant reservoir,” effectively shielding the bacteria from the host’s immune response and most conventional antibiotics [15,16]. Upon maturation, bacteria can be released from these niches to re-infect the bladder, leading to clinical relapses [15]. These biofilms create a resilient barrier that further complicates treatment by preventing the effective diffusion of antimicrobial agents [15].

## Review

Methodology of the review

This manuscript presents a structured narrative literature review on the management and prevention of rUTIs in women, focusing on publications between 2022 and 2026 [1,12]. This period reflects a contemporary shift in clinical practice toward reducing long-term antibiotic use and increasing reliance on evidence-based non-antimicrobial strategies [1,3,10]. The review integrates findings from international clinical guidelines, meta-analyses, and pivotal clinical trials to provide a comparative overview of global and national recommendations [4,5,12].

Literature Search and Identification

A focused literature search was performed to identify relevant clinical evidence and updated practice guidelines [4,6]. The search strategy was concluded in July 2026 and was limited to peer-reviewed publications in English and Polish to facilitate a comprehensive comparison between international standards and local clinical practice. The following databases were consulted: PubMed/MEDLINE, EMBASE, the Cochrane Library, Scopus, and Google Scholar [1,4,6]. Searches combined Medical Subject Headings (MeSH) terms and free-text keywords to identify pertinent publications [4,9]. Primary condition: “Recurrent urinary tract infection” OR “rUTI” OR “recurrent cystitis.” Strategy and policy: “Non-antibiotic prophylaxis” OR “antimicrobial stewardship” OR “antibiotic-sparing” OR “prevention.” Specific agents: “methenamine hippurate,” “D-mannose,” “vaginal estrogen,” “immunoactive prophylaxis,” “bacterial vaccines,” and “phage therapy.”

Inclusion and Exclusion Criteria

Study selection followed a two-stage screening process. Initially, titles and abstracts were independently screened by two reviewers to identify potentially relevant data. In the second stage, full-text articles were assessed for eligibility based on the PICOTS framework, with any discrepancies resolved through consensus. Inclusion criteria were studies involving adult women with a verified diagnosis of rUTI (defined as ≥2 symptomatic episodes in 6 months or ≥3 in 12 months) [1,13], clinical practice guidelines from major international and national urological and gynecological societies (EAU, AUA/Canadian Urological Association (CUA)/Society of Urodynamics, Female Pelvic Medicine & Urogenital Reconstruction (SUFU), National Institute for Health and Care Excellence (NICE), Polish Urological Association (PTU), Polish Society of Gynecologists and Obstetricians (PTGiP), Polish Society of Family Medicine (PTMR)) [4,11], randomized controlled trials (RCTs), meta-analyses, and high-quality observational studies focusing on contemporary prevention strategies [4,6]. Exclusion criteria were studies focusing exclusively on pediatric populations (<16 years) or pregnant women [4,7], patients with anatomical or functional urinary tract abnormalities [1,4], non-peer-reviewed materials, conference abstracts without full datasets, and studies lacking direct clinical applicability [4,8].

Quality Assessment and Evidence Grading

Instead of performing a formal systematic risk-of-bias assessment, this review relied on a qualitative appraisal of the strength and clinical relevance of the included evidence. Particular emphasis was placed on recommendations from established clinical practice guidelines, which already incorporate evidence grading frameworks such as Grading of Recommendations Assessment, Development and Evaluation (GRADE) or Oxford Centre for Evidence-Based Medicine (CEBM) levels [6,7].

Data Synthesis and Consensus

An initial search across the selected databases identified 6,231 records. After removing duplicates and screening for relevance according to the specified criteria, 28 key source documents, including major clinical guidelines and high-impact clinical trials, were selected for the final analysis. The final evidence base was selected to prioritize studies with the highest impact on antimicrobial stewardship. A narrative synthesis approach was employed to compare international practice standards with current Polish national guidelines [11,12]. In areas where high-quality prospective data remain sparse (a common issue in rUTI management), this review incorporates Clinical Principles and Expert Opinions derived from the WikiGuidelines Group (2024) and recent society consensus panels [14]. This ensures that the review captures not only statistical efficacy but also “real-life” clinical utility and patient-centered perspectives [14].

Diagnostics of recurrent urinary tract infections

The diagnosis of rUTIs requires a rigorous approach to ensure clinical accuracy and avoid the over-prescription of antimicrobials [1,5,11].

Clinical Presentation and Symptoms

A clinical diagnosis is primarily established based on typical lower urinary tract symptoms (LUTS) [7,11,12]. These include dysuria (pain, burning, or stinging), frequency, urgency, and suprapubic pain [5,13,14]. The presence of these symptoms in the absence of vaginal discharge or irritation provides a high probability of uncomplicated cystitis [1,5,13]. However, clinical presentation varies significantly with age [2,3,5]. While reproductive-age women typically present with localized LUTS, elderly or frail patients may exhibit atypical symptoms, such as delirium, sudden functional decline, falls, or malaise, which are not always directly localized to the urinary tract [1,2,5].

Diagnostic Criteria and the Role of Microbiological Confirmation

While an isolated episode of uncomplicated cystitis may be treated based on symptoms alone, guidelines now strongly emphasize that every episode of suspected rUTI must be confirmed by urine culture [1,7,11]. This is essential to establish the infectious etiology, differentiate between reinfection (a new infection with different bacteria) and relapse (persistence of the same pathogen within 14 days), and guide targeted, narrow-spectrum therapy [2,7,13]. The microbiological threshold for a positive culture in symptomatic women is widely accepted as ≥103 colony-forming units (CFU)/mL of a known uropathogen [5,11,13]. In contrast, asymptomatic bacteriuria (ABU) is defined by bacterial growth of ≥105 CFU/mL in two consecutive samples in women who do not exhibit any urinary tract symptoms [1,11,12]. Distinguishing between rUTI and ABU is critical, as current guidelines recommend against screening or treating ABU in non-pregnant, non-surgical patients to prevent “collateral damage” to the host microbiome and the selection of resistant strains [1,5,11].

Differential Diagnosis

Clinicians must exclude conditions with overlapping symptomatic profiles, including [5,7,13] overactive bladder, characterized by urgency and frequency without evidence of infection [5,7,16]; interstitial cystitis/painful bladder syndrome, characterized by chronic pelvic pain and LUTS with no identifiable infection [5,12,13]; atrophic vaginitis, common in postmenopausal women, where estrogen deficiency causes vaginal dryness and irritation that mimics UTI [5,9,13]; sexually transmitted infections, urethritis caused by *Chlamydia trachomatis* or *Mycoplasma* spp. should be suspected in sexually active patients presenting with pyuria but negative standard urine cultures [5,11,12].

Role of Imaging and Invasive Testing

Extensive routine workups, such as cystoscopy or full abdominal ultrasound, are not indicated for otherwise healthy women under the age of 40 with no specific risk factors, as the diagnostic yield is low [1,4]. However, imaging and invasive procedures are recommended in atypical or complicated cases [12,13] such as suspicion of urolithiasis or anatomical abnormalities, persistent gross hematuria or a history of smoking (to rule out urothelial cancer), recurrent infections caused by *Proteus* spp., which are frequently associated with struvite stones, and a history of upper tract infections or prior urological surgery. A physical examination should include a pelvic assessment to identify modifiable structural abnormalities such as cystocele, pelvic organ prolapse, or significant vaginal atrophy [7,13]. Furthermore, assessing the post-void residual (PVR) volume via ultrasound is recommended, as a PVR >150 mL is an abnormal finding that predisposes the patient to bacterial proliferation and recurrence [12,13].

Current management guidelines (2023-2026): A comparative analysis

Contemporary management of rUTI is defined by a global commitment to prioritize non-antibiotic preventive strategies over long-term suppressive therapy [5,14].

European Association of Urology 2024/2026 Recommendations

The latest EAU updates emphasize a stepwise approach: behavioral modifications, followed by non-antimicrobial measures, and, finally, antibiotic prophylaxis [1,12]. Regarding non-antibiotic strategies, strong recommendations are provided for immunoactive prophylaxis (OM-89/Uro-Vaxom) and vaginal estrogen replacement in postmenopausal women [1,4,12]. For antibiotic-sparing agents, the 2026 guidelines include a strong recommendation for methenamine hippurate (1 g twice daily) as an effective alternative for women without anatomical abnormalities [12]. Weak recommendations are given for D-mannose and cranberry products due to clinical evidence heterogeneity, requiring clinicians to fully inform patients of the contradictory data [1,12].

American Urological Association/Canadian Urological Association/Society of Urodynamics, Female Pelvic Medicine & Urogenital Reconstruction 2025 Amendments

The combined American and Canadian guidelines align closely with the EAU on definitions and the necessity of culture-proven episodes [4,7]. They highlight the importance of shared decision-making and patient education regarding the risks of long-term antibiotic use, such as *Clostridioides difficile* infection and increased resistance [3,7]. The AUA also supports the use of cranberry supplements as an economical preventive method, specifically noting the FDA-validated health claim for cranberry products in reducing UTI risk [7].

Polish National Guidelines (Polish Urological Association, Polish Society of Gynecologists and Obstetricians, Polish Society of Family Medicine) 2024

The summary of Polish guidelines (2024) reflects regional characteristics in etiology and drug availability [11]. As a first-line antimicrobial selection for acute uncomplicated cystitis, the recommended agents include fosfomycin trometamol, pivmecillinam, nitroxoline, and furazidin (furagin), the latter being a specific over-the-counter option widely used in Poland [11]. For continuous prevention, Polish experts suggest fosfomycin (3 g every 10 days), trimethoprim, or furazidin [11]. Like their international counterparts, they emphasize the clinical significance of behavioral management, vaginal probiotics, and immunoprophylaxis [11].

WikiGuidelines Group Consensus Statement (2024)

The WikiGuidelines Group provides a distinct, evidence-based perspective published in JAMA Network Open. Their methodology is more restrictive, only issuing “clear recommendations” when supported by high-quality, reproducible prospective data [14]. Confirmed effective: They issue clear recommendations for vaginal estrogen, methenamine hippurate, and cranberry products (standardized to 36 mg of proanthocyanidins) [14]. Inconclusive: Probiotics and increased water intake (for non-geriatric populations) are categorized under “clinical review” due to the need for more confirmatory studies.

A comparative analysis of international and national clinical guidelines for the management of recurrent urinary tract Infections (2024-2026) is presented in Table 1.

**Table 1:** Comparative analysis of international and national clinical guidelines for the management of recurrent urinary tract infections (2024–2026). AUA/CUA/SUFU: American Urological Association/Canadian Urological Association/Society of Urodynamics, Female Pelvic Medicine & Urogenital Reconstruction; C. difficile: *Clostridioides difficile*; EAU: European Association of Urology; PAC: Proanthocyanidins; Polish National Guidelines: Joint recommendations by the Polish Urological Association (PTU), Polish Society of Gynecologists and Obstetricians (PTGiP), and Polish Society of Family Medicine (PTMR)

Category/Intervention	EAU (2024/2026) [1,4,12]	AUA/CUA/SUFU (2025) [3,4,7]	Polish National Guidelines (2024) [11]	WikiGuidelines Group (2024) [14]
General strategic approach	Stepwise management: Behavioral modifications, non-antimicrobial measures, antibiotic prophylaxis	Shared decision-making: Focus on patient education regarding risks of long-term antibiotics (e.g., *C. difficile*).	Regional adaptation: Focus on local etiology and specific drug availability (e.g., furazidine)	Evidence-restrictive: Clear recommendations issued only when supported by high-quality prospective data
Vaginal estrogen therapy	Strong recommendation for postmenopausal women	Supported as a key preventive measure	Clinical significance emphasized by national experts	Confirmed effective: Clear recommendation for use
Methenamine hippurate	Strong recommendation (1 g twice daily) for patients without anatomical abnormalities	Supported as an effective antimicrobial-sparing alternative	Not explicitly detailed in the provided prophylaxis section	Confirmed effective: Clear recommendation for use
Immunoactive prophylaxis	Strong recommendation for OM-89 (Uro-Vaxom)	Included as a supported prevention strategy	Emphasized as clinically significant	Not listed as “confirmed effective” in the provided consensus
Cranberry products	Weak recommendation due to clinical evidence heterogeneity	Supported: Recognized as an economical method with FDA-validated health claims	Not explicitly detailed in the provided prophylaxis section	Confirmed effective: Specifically when standardized to 36 mg PAC
D-mannose	Weak recommendation: Clinicians must inform patients of contradictory data	Not explicitly detailed in the provided text	Not explicitly detailed in the provided text	Inconclusive: Categorized under “clinical review”; requires more data
Continuous antibiotic prophylaxis	Recommended only as a final step in the hierarchy of care	Cautioned against due to risk of “collateral damage” and resistance	Recommended agents: Fosfomycin (3 g/10 days), trimethoprim, or furazidine	Not detailed in the provided evidence-based summary
Diagnostic framework	Adheres to standard temporal definitions (2/6 or 3/12 months)	Mandatory microbiological confirmation of every suspected episode	Focus on differentiating reinfection from relapse via culture	Terminology Shift: Advocates for “localized vs. systemic” instead of “uncomplicated”

Established evidence-based non-antibiotic prophylaxis (standard of care)

There is a weak but consistent recommendation for women to increase their daily fluid intake by an additional 1.5 L [1,7,12]. Clinical trials demonstrate that this strategy significantly reduces UTI frequency in women who previously consumed less than 1.5 L/day [7,12]. Post-coital micturition and proper menstrual hygiene are frequently recommended to facilitate the mechanical clearance of pathogens from the urethra [11,12]. Clinicians should focus on evidence-based advice to avoid stigmatizing patients with non-validated “hygiene” mandates [7,9]. In postmenopausal women, vaginal estrogen replacement receives a strong recommendation [1,12]. Estrogen deficiency leads to the depletion of protective *Lactobacillus* species and an increase in vaginal pH, predisposing the urothelium to colonization by UPEC [2,9].

Therapeutic Strategies

For formulations and dosing, effective regimens include [5,7] estrogen cream (0.1 mg/g) applied nightly for two weeks, then twice weekly; vaginal suppositories (4 or 10 µg) administered nightly for two weeks, then twice weekly; and vaginal rings (7.5 µg/24 hours) replaced every three months. Topical application has been shown to restore the vaginal microbiome and significantly reduce recurrence rates compared to placebo, although it may be inferior to continuous antibiotic prophylaxis (CAP) [7,12]. Oral estrogen therapy is not recommended due to lack of efficacy and an unfavorable side effect profile [2,7,12].

Immunomodulatory agents aim to stimulate the host’s innate and adaptive immune responses against common uropathogens [6,7,16-21]. Oral OM-89 (Uro-Vaxom): This lyophilized extract of 18 *E. coli* strains is strongly recommended by the EAU for all age groups [1,16]. The standard regimen is one 6 mg capsule daily for 90 days [8,16]. Clinical data indicate it can reduce UTI recurrences by 34% to over 80% [16].

Methenamine hippurate serves as a powerful non-antibiotic alternative, hydrolyzed to bactericidal formaldehyde in acidic urine [5,9,12]. The standard dose is 1 g twice daily [1,7,12]. The ALTAR trial (2022) established its non-inferiority to low-dose antibiotic prophylaxis in women with rUTI, with the added benefit of significantly lower rates of AMR development [7,12,22]. The ImpresU trial (2025) further confirmed its efficacy in women aged ≥70, showing a 25% reduction in antibiotic treatments for UTI [23]. It is now considered a strong first-line alternative for women without anatomical urinary tract abnormalities [1,7,12,23].

Antibiotic prophylaxis strategies

While modern guidelines prioritize non-antibiotic interventions, antimicrobial prophylaxis remains the most effective strategy for reducing recurrences when other measures have failed [5,13].

Post-coital Prophylaxis

Post-coital prophylaxis is highly effective for women whose UTI episodes are temporally related to sexual activity [7,12]. A single dose administered immediately after intercourse can reduce the risk of infection without the cumulative side effects of daily medication [5,7]. Recommended regimens are nitrofurantoin 50 mg or 100 mg [7,11], trimethoprim-sulfamethoxazole 40/200 mg or 80/400 mg [7], cephalexin 250 mg [7,11], and furazidine (Polish-specific) 50 mg [11].

Continuous Low-Dose Prophylaxis

CAP is reserved for patients with high-frequency recurrences who do not respond to behavioral or non-antimicrobial strategies [7,11,12]. The typical duration of therapy ranges from 3 to 12 months, followed by reassessment [7,12]. Agent selection and dosing are nitrofurantoin 50 mg or 100 mg once daily (a preferred choice due to low resistance rates in *E. coli*) [7,11,12], fosfomycin trometamol 3 g every 7 to 10 days [11,12], trimethoprim 100 mg once daily [7,11,12], and cefaclor/cephalexin 250 mg once daily (often used in pregnancy) [11,12]. Systematic reviews indicate that CAP can reduce UTI episodes by up to 95% during treatment; however, recurrences frequently occur once the medication is discontinued [7,9,12,13].

Self-start (Patient-initiated) Therapy

For motivated and well-educated patients, self-start therapy offers a safe and economical alternative to continuous suppression [7,12]. Patients are provided with a prescription to initiate a standard short-course treatment at the first sign of typical symptoms [7,12]. Guidelines suggest obtaining a urine culture before starting the dose whenever possible to monitor for resistance [7,11].

Adjuvant and emerging biological therapies (evolving evidence)

In cases where conventional behavioral and pharmacological strategies fail to control rUTI, advanced local therapies and emerging biological interventions offer promising alternatives [3]. While the following interventions show clinical potential, it is important to distinguish them from established standards of care [3]. These therapies are currently supported by preliminary clinical series or emerging evidence rather than high-quality, multicenter guideline recommendations, and should be considered primarily for refractory cases or within research settings [3]. These methods focus on direct urothelial restoration, the use of biological predators to combat multidrug-resistant (MDR) pathogens, and the enhancement of regenerative pathways [15].

Advanced Immunoprophylaxis 

FimCH vaccine: An emerging investigational vaccine targeting the FimH adhesin of UPEC [3]. Early clinical series demonstrate its potential to induce long-term antibody responses and significantly reduce recurrences in high-risk patients, but it remains in the investigational phase [17,18].

Sublingual MV140 (Uromune): A polyvalent bacterial preparation (containing inactivated strains of *E. coli*,* Klebsiella pneumoniae*, *Proteus vulgaris*, and *Enterococcus faecalis*) administered sublingually for three months [10]. Recent phase II and real-life studies report UTI-free rates of up to 38% at 12 months, with an overall reduction in UTI frequency of approximately 67-82% [19,20]. The EAU 2026 guidelines have assigned it a “Weak” recommendation status, citing the need for more large-scale, independent RCTs to confirm long-term efficacy [1,4,12].

Dietary Supplements and Phytotherapy

The efficacy of dietary supplements varies, often requiring shared decision-making to manage patient expectations regarding contradictory evidence [1,5,12].

D-mannose: It is traditionally used to prevent the attachment of type 1 fimbriated *E. coli* to the urothelium [2,3,5]. While older studies suggested parity with nitrofurantoin, the landmark MERIT trial (2024) found that 2 g daily dosing did not significantly reduce medically attended UTIs in a primary care setting [2,12,21]. Current recommendations are generally weak due to this clinical heterogeneity [1,12].

Cranberry products: Standardized extracts containing at least 36 mg of proanthocyanidins are recommended to prevent bacterial adhesion [5,14]. While meta-analyses are conflicting, high-dose proanthocyanidins (240 mg) or standardized phytosomes have shown success in specific populations, such as postmenopausal women with diabetes [7,12].

Phytotherapy (Canephron N): This is a combination of *Centaurii herba*, *Levistici radix*, and *Rosmarini folium* [12]. Clinical trials indicate it is effective in relieving acute symptoms and reducing the frequency of recurrent episodes over 6 to 12 months, but high-quality evidence for long-term prophylaxis is still evolving [12].

Intravesical Instillations: Glycosaminoglycan Layer Replenishment

The bladder’s glycosaminoglycan (GAG) layer, composed of hyaluronic acid (HA), chondroitin sulfate (CS), heparin sulfate, and dermatan sulfate, serves as a critical primary barrier against bacterial adhesion [4,7,17]. Chronic inflammation and repeated infections often lead to GAG layer deficits, facilitating the attachment of UPEC to the underlying urothelium [4,7].

Hyaluronic acid and chondroitin sulfate: Endovesical instillations of HA alone or in combination with CS are strongly recommended for patients who have failed less invasive preventive approaches [4,12]. Meta-analyses involving both RCTs and observational studies have demonstrated a significant reduction in the pooled mean rate of UTIs per year and a significantly longer mean time until the first recurrence [4,12].

Regimen and efficacy: While protocols vary, treatment typically spans two to six months, with a total follow-up of 12 to 18 months showing sustained clinical benefits [12]. Patients should be informed that while initial results are favorable, large-scale studies are still needed to standardize dosing and confirm long-term outcomes [1,12].

Intravesical antimicrobials: Emerging research investigates the direct administration of antibiotics, such as gentamicin, via dedicated catheters [3,24]. This approach achieves high local concentrations at the apical urothelial surface, facilitating the penetration of the urothelial barrier to eradicate pathogens residing in deeper layers while minimizing systemic absorption and the risk of developing AMR [3,24].

Emerging Biological and Regenerative Therapies

Phage therapy: Bacteriophages are viruses that specifically target and lyse pathogenic bacteria, offering a tailored approach to treating MDR infections [14,25]. Systematic reviews of case reports, including collaborations between the Medical University of Warsaw and the Hirszfeld Institute of Immunology (Poland), highlight the successful use of intrarectal or intravesical phage cocktails against extended-spectrum beta-lactamase-producing *Klebsiella pneumoniae* [25]. Challenges include the time-intensive nature of performing a phagogram and the necessity of using polyvalent cocktails to prevent the emergence of phage-resistant bacterial mutants [25].

Platelet-rich plasma (PRP): PRP is an autologous blood-derived product containing high concentrations of growth factors, such as platelet-derived growth factor, transforming growth factor-beta, and vascular endothelial growth factor, which facilitate tissue repair and angiogenesis [26]. In patients with intractable rUTI, repeated intravesical PRP injections have been shown to enhance the expression of barrier function proteins such as E-cadherin and ZO-1, and increase the proliferative marker Ki-67, thereby restoring urothelial integrity [17,26]. Clinical trials suggest that PRP reduces the frequency of infection episodes and improves functional bladder capacity [26].

Antimicrobial peptides (AMPs): AMPs are evolutionarily conserved cationic peptides that kill bacteria by disrupting their membranes or inhibiting intracellular transcription and translation [3,14]. Several AMPs, including lactoferrin and defensins, are being explored as monotherapies or in synergy with traditional antibiotics to enhance bactericidal activity and disrupt biofilms [3].

Fecal microbiota transplantation (FMT): Preliminary data suggest that FMT, by restoring intestinal microbiome diversity, can reduce the fecal reservoir of uropathogenic Enterobacteriaceae, potentially decreasing the incidence of recurrent ascending UTIs [3].

A comparison of the dosage and clinical strength of contemporary antibiotic-sparing treatments is presented in Table 2.

**Table 2:** Detailed non-antibiotic prophylactic options: A comparison of the dosage and clinical strength of contemporary antibiotic-sparing treatments. E-cadherin: epithelial cadherin; EAU: European Association of Urology; GAG: glycosaminoglycans; HA/CS: hyaluronic acid/chondroitin sulfate; MV140: sublingual polyvalent bacterial vaccine (Uromune®); OM-89: oral *Escherichia coli* lysate (Uro-Vaxom®); PRP: platelet-rich plasma; rUTI: recurrent urinary tract infection

Intervention	Route and dosage	Evidence strength	Clinical pearl/Key insight
Methenamine hippurate [1,5,7,9,12,22,23]	1 g twice daily	Strong (clear)	Converts to formaldehyde in acidic urine; effective alternative to prophylactic antibiotics
Vaginal estrogen [1,2,5,7,9,12]	Cream, suppository, or ring	Strong (clear)	Restores protective *Lactobacillus* flora and lowers vaginal pH
(Uromune®) MV140 [10,19,20]	2 sublingual sprays daily for 3 months	Promising	Polyvalent vaccine (4 species); reported UTI-free rates of up to 58–90% at 12 months
OM-89 (Uro-Vaxom®) [1,8,16]	6 mg oral capsule daily for 90 days	Strong (EAU)	Lyophilized *E. coli* lysate; reduces recurrences by 34% to >80%
GAG instillations (HA/CS) [4,7,12,17]	Periodic endovesical instillations	Weak	Replenishes the GAG layer; used for refractory cases
PRP [17]	Repeated intravesical injections	Emerging	Stimulates cell proliferation (Ki-67) and barrier protein expression (E-cadherin)
Vaginal lubrication [1,7,12]	Water/Silicone-based (no spermicides)	Mechanical support	Prevents urethral micro-trauma during sex, which otherwise facilitates bacterial adhesion
Increased hydration [1,7,12]	Additional 1.5 L daily	Weak	Mechanical “flushing” of bacteria; simple and safe for premenopausal women

Discussion: Challenges in clinical practice

UPEC remains the primary causative agent in approximately 80% of rUTI cases [3,6]. The global escalation of AMR represents a critical threat, with resistance rates for UPEC to routinely prescribed antibiotics, such as fluoroquinolones, reaching as high as 70% in certain regions [3]. Furthermore, up to 60% of UPEC strains now express extended-spectrum β-lactamases, complicating outpatient management and often requiring intravenous interventions [3,27,28].

Personalization of Treatment: Risk Factor Identification

Successful rUTI management requires a move away from “one-size-fits-all” approaches toward the identification and mitigation of modifiable host-related risk factors [1-2]. Structural issues such as pelvic organ prolapse and cystoceles frequently result in significant PVR urine volume [1,3,4]. Incomplete bladder emptying provides a stagnant environment favorable for bacterial proliferation [1,5]. Metabolic conditions (diabetes) due to hyperglycemia and glycosuria create a microbial-rich niche, while impaired insulin signaling may reduce the production of endogenous AMPs [17]. A maternal history of UTI and the occurrence of a first infection before the age of 15 are independent risk factors for recurrence [1,4,5]. Furthermore, polymorphisms in Toll-like receptors, which are essential for recognizing pathogens in the urinary tract, can impair the host’s innate immune response [5].

Shared Decision-Making and Patient Education

Given the diverse range of prevention strategies and varying patient goals, shared decision-making has become a clinical standard [1,8]. Clinicians should engage patients in a collaborative partnership to balance immediate symptom relief and repetitive antimicrobial therapy [1,8]. Many women suffer from a constant fear of a new infection [9,20]. Clinicians can reduce this psychological burden by explaining that localized cystitis is typically self-limiting and rarely progresses to pyelonephritis or systemic illness [5,7]. Providing evidence-based options, such as topical vaginal estrogen for postmenopausal women or bacterial vaccines (OM-89/MV140), empowers patients to choose sustainable long-term strategies over daily antibiotic suppression [1,9,16,20]. However, clinicians must also contextualize the economic burden of self-care; for example, D-mannose is a popular non-prescription supplement, yet its high monthly cost and recent evidence from the landmark MERIT trial (2024) challenge its routine efficacy in primary care [2,12,21].

Superiority of Non-antimicrobial Alternatives

Methenamine hippurate (1 g twice daily) and topical vaginal estrogen (for postmenopausal women) represent the strongest non-antibiotic preventive options [1-3]. Landmark clinical trials such as ALTAR and ImpresU have established that methenamine hippurate is non-inferior to low-dose antibiotic prophylaxis while significantly reducing the selection pressure for resistance. Therapeutic management should follow a clear, stepwise hierarchy: initially focusing on behavioral and lifestyle modifications, followed by non-antimicrobial prophylaxis, and reserving continuous or post-coital antibiotic suppression as a last-line measure [22,23].

Emerging and Adjuvant Therapies

For patients with “intractable” rUTI who fail conventional measures, intravesical instillations of HA and CS provide effective GAG layer replenishment [4,12]. Furthermore, biological predator strategies such as phage therapy and regenerative treatments such as PRP show substantial promise in early clinical series and represent the future frontier of personalized urological care [24-26]. A critical evidence gap remains regarding complex populations, such as those with indwelling catheters, as most current trials exclude these high-risk groups. Future research must prioritize large-scale, independent RCTs to evaluate the synergistic potential of phage-antibiotic combinations for these unaddressed cohorts [4]. A hierarchy of preventive management for rUTI is presented in Table 3.

**Table 3:** Hierarchy of preventive management for rUTI. GAG: glycosaminoglycans; HA/CS: hyaluronic acid/chondroitin sulfate; MV140: sublingual polyvalent bacterial vaccine (Uromune®); OM-89: oral *Escherichia coli* lysate (Uro-Vaxom®); PRP: platelet-rich plasma; rUTI: recurrent urinary tract infection Compiled by the authors based on the European Association of Urology Guidelines 2024–2026, American Urological Association/Canadian Urological Association/Society of Urodynamics, Female Pelvic Medicine & Urogenital Reconstruction Guidelines 2025, and specific clinical evidence from the ALTAR, ImpresU, and MERIT trials [1-4,7,11,12,21-23]. This table represents an original synthesis of current clinical data; no copyrighted material was reproduced or adapted.

Step	Category	Recommended interventions
1	Behavioral and mechanical	Increased fluid intake (additional 1.5 L/day); Vaginal lubrication/moisturization before intercourse (to reduce mechanical friction); voiding after sex
2	Hormonal therapy	Topical vaginal estrogen replacement for postmenopausal women (strong recommendation)
3	Immunoactive prophylaxis	Oral OM-89 (Uro-Vaxom®) or sublingual MV140 (Uromune®) to stimulate trained mucosal immunity
4	Non-antibiotic antiseptics	Methenamine hippurate (1 g twice daily); non-inferior to antibiotics with no resistance induction
5	Advanced local therapy	Intravesical instillations (GAG layer replenishment with HA/CS) for patients who fail less invasive measures
6	Regenerative therapy	Intravesical PRP injections to stimulate urothelial repair and restore barrier function
7	Antibiotic prophylaxis	Reserved as a last resort: post-coital (single dose) or continuous low-dose suppression

Discrepancies Between Guidelines

The observed discrepancies between major clinical guidelines reflect fundamental differences in methodological frameworks and evidence grading thresholds. For instance, the WikiGuidelines Group (2024) restricts its recommendations strictly to areas supported by high-quality prospective data, whereas societies such as the EAU and AUA/CUA/SUFU provide pragmatic guidance through Expert Opinions or Clinical Principles to address clinical gaps where RCTs are sparse [1,3,4,7,14]. Furthermore, discrepancies often arise from the variable regulatory status and availability of interventions; for example, the sublingual vaccine MV140 is widely utilized in Europe but lacks current approval in North America, while methenamine hippurate remains unavailable in markets such as Germany [1,3,4,7,14]. Finally, national guidelines, such as the Polish Summary (2024), are specifically tailored to regional AMR patterns and local drug access, such as the widespread clinical use of furazidine, ensuring that management remains contextually relevant to the local population [11].

Limitations

This review has several limitations. As a narrative review, it may be subject to selection and interpretation bias, and no formal risk-of-bias assessment was performed. Furthermore, the possibility of publication bias cannot be excluded. These limitations should be considered when interpreting the findings and recommendations presented in this review.

## Conclusions

The management of rUTI in women is undergoing a fundamental shift from the repetitive treatment of acute symptomatic episodes to a long-term, preventive, and antibiotic-sparing strategy. This transition is necessitated by the global crisis of AMR and the recognition of rUTI as a chronic condition that significantly impairs health-related quality of life. Accurate diagnosis, confirmation of symptomatic infections, and avoidance of unnecessary antimicrobial treatment, particularly in asymptomatic bacteriuria, are essential components of effective management. Therapeutic management should follow a clear, stepwise hierarchy: initially focusing on behavioral and lifestyle modifications, followed by non-antimicrobial prophylaxis, and reserving continuous or post-coital antibiotic suppression as a last-line measure. Despite advancements, a significant gap remains between clinical practice and evidence-based guidelines. Future research must focus on high-quality, large-scale randomized trials to standardize dosing for bacterial vaccines and nutraceuticals, ensuring that management moves away from “eminence-based” practice toward true evidence-based clinical principles.
